# Reactivation of Latent Tuberculosis Following COVID-19 and Epstein-Barr Virus Coinfection: A Case Report

**DOI:** 10.3390/pathogens14050488

**Published:** 2025-05-16

**Authors:** Iryna Halabitska, Pavlo Petakh, Valentyn Oksenych, Oleksandr Kamyshnyi

**Affiliations:** 1 Department of Therapy and Family Medicine, I. Horbachevsky Ternopil National Medical University, Voli Square, 1, 46001 Ternopil, Ukraine; 2 Department of Biochemistry and Pharmacology, Uzhhorod National University, 88000 Uzhhorod, Ukraine; pavlo.petakh@uzhnu.edu.ua; 3 Broegelmann Research Laboratory, Department of Clinical Science, University of Bergen, 5020 Bergen, Norway; 4 Department of Microbiology, Virology, and Immunology, I. Horbachevsky Ternopil National Medical University, 46001 Ternopil, Ukraine

**Keywords:** COVID-19, Epstein–Barr virus, *Mycobacterium tuberculosis*, lymphopenia, lymphadenopathy, scrofuloderma

## Abstract

**Background:** This case is unique in demonstrating the reactivation of latent tuberculosis (TB) following co-infection with SARS-CoV-2 and Epstein–Barr virus (EBV) in an otherwise healthy young adult. It highlights a rare clinical scenario in which viral immune dysregulation likely facilitated TB progression. To date, few reports have explored the complex interplay between COVID-19, EBV reactivation, and TB in a single patient, particularly with isolated extrapulmonary involvement. **Case Presentation:** A 24-year-old woman presented with persistent low-grade fever, fatigue, night sweats, unintentional weight loss, and progressive cervical and supraclavicular lymphadenopathy. These symptoms emerged shortly after a moderate COVID-19 infection. Laboratory studies revealed elevated inflammatory markers and pronounced lymphopenia. EBV reactivation was confirmed via serology and PCR. Despite antiviral therapy, symptoms persisted, and imaging revealed necrotic lymphadenopathy. Tuberculous lymphadenitis was diagnosed through fine-needle aspiration cytology and PCR detection of *Mycobacterium tuberculosis*. The patient was treated with a standard anti-tuberculosis regimen, resulting in clinical, radiological, and immunological improvement. **Conclusions:** This case underscores the importance of considering latent TB reactivation in patients with persistent lymphadenopathy and recent viral infections, particularly in regions with high TB prevalence. It also emphasizes the need for thorough immunological and microbiological assessment in complex post-viral syndromes. The main clinical takeaway is that COVID-19 and EBV co-infection may create a permissive environment for TB reactivation through immune system compromise.

## 1. Introduction

Tuberculous lymphadenitis (scrofuloderma) is the most common form of extrapulmonary tuberculosis, presenting as chronic lymphadenopathy that can mimic a wide range of infectious, autoimmune, and neoplastic diseases [1,2]. The diagnostic challenge is further compounded by overlapping clinical manifestations with systemic illnesses, particularly viral infections such as COVID-19 and Epstein–Barr virus (EBV) [3,4,5]. Both viruses can cause prolonged lymphadenopathy, immune dysfunction, and lymphopenia, complicating the determination of the underlying etiology [6,7].

EBV is a well-known trigger of lymphoproliferative disorders and can cause infectious mononucleosis, characterized by generalized lymphadenopathy, hepatosplenomegaly, and immune disturbances [8,9]. At the same time, COVID-19, beyond its respiratory manifestations, has been associated with persistent immune alterations, including lymphocyte depletion, which may increase susceptibility to secondary bacterial and mycobacterial infections [10,11,12,13].

Given the epidemiological significance of tuberculosis and the growing role of viral infections in immune system modulation, maintaining a high level of clinical suspicion is crucial when evaluating patients with persistent lymphadenopathy [14,15]. In this context, timely differential diagnosis between tuberculous lymphadenitis, viral infections, and other systemic diseases is essential for appropriate treatment and improved patient outcomes [16,17]. Other causes of lymphadenopathy include bacterial infections such as *Bartonella henselae* or *Brucella* spp., parasitic diseases like toxoplasmosis, autoimmune conditions including systemic lupus erythematosus and sarcoidosis, and malignancies such as lymphoma and leukemia [17,18,19]. Differentiating among these conditions requires a systematic approach combining clinical examination with imaging, laboratory tests, and histopathological evaluation of lymph node tissue [20,21]. Awareness of the full spectrum of potential etiologies is critical to avoid diagnostic delays and ensure timely, targeted management [22].

## 2. Case Description

### 2.1. Patient Information

A 24-year-old female presented with a subacute onset of systemic and localized symptoms following a documented episode of moderate COVID-19, which occurred three months prior to consultation. Her primary complaints included persistent low-grade fever, fatigue, generalized lymphadenopathy, unintentional weight loss, and night sweats, with a particularly concerning development of a painful, bluish supraclavicular mass two days before presentation. The patient had no prior history of tuberculosis, immunodeficiency, or chronic illness, and no relevant family history of infectious or autoimmune diseases was reported. She did not use immunosuppressive therapy prior to her COVID-19 treatment and had no significant psychosocial or environmental risk factors for tuberculosis. Immunological evaluation revealed substantial lymphopenia, including reductions in CD4+ and CD8+ T cells, B cells, and NK cells, alongside low levels of complement C4 and immunoglobulins, indicating notable immune dysregulation.

### 2.2. Clinical Findings

**Figure 1:**
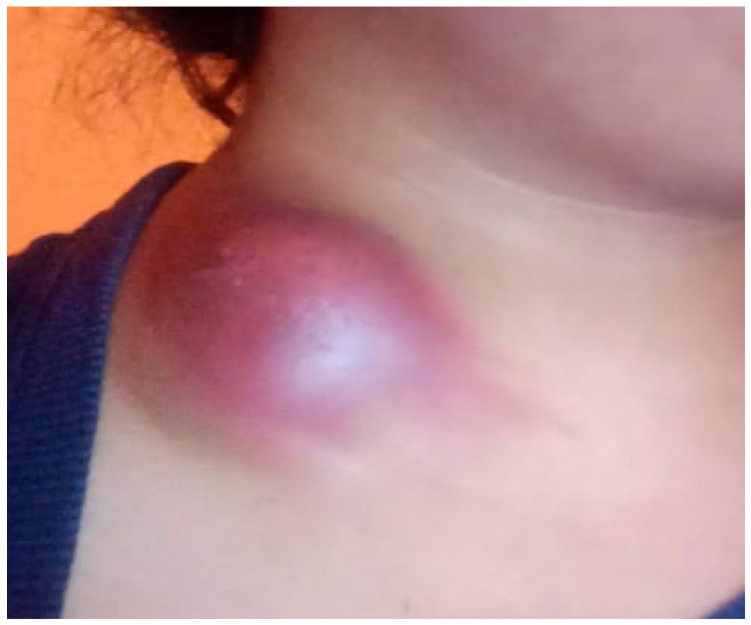
**Acute subcutaneous inflammatory mass in the neck region.** The image shows the clinical presentation observed in the patient, which includes a pathological mass on the anterolateral surface of the neck with marked localized hyperemia, edema, and a dome-shaped tissue protrusion. The skin in this area appears taut and glossy, indicating increased intratissue pressure.

On presentation, physical examination revealed a raised, bluish mass over the left supraclavicular region, accompanied by localized skin erythema, tension, and mild tenderness. The lesion demonstrated inflammatory features including edema and a dome-shaped protrusion. Palpation identified multiple firm, mobile, non-tender lymph nodes in both cervical and supraclavicular areas, with some forming clusters. No hepatosplenomegaly or signs of respiratory involvement were observed. Vital signs were stable, although the patient continued to report fever and a marked decline in physical stamina. General examination was otherwise unremarkable, aside from signs of anemia (Figure 1).

### 2.3. Timeline

**Figure 2:**
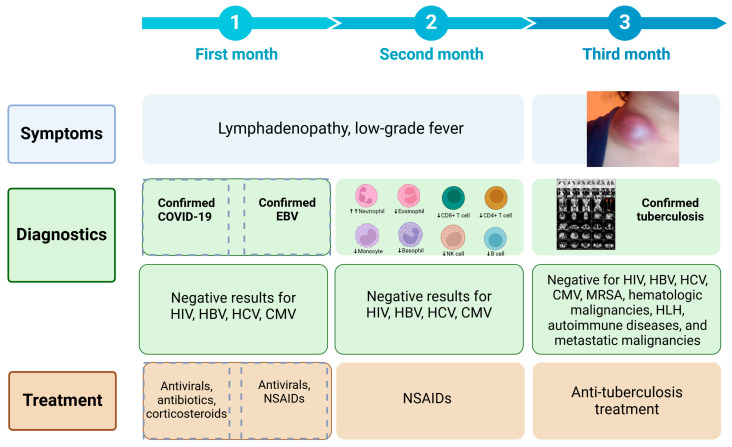
Timeline of symptom progression, diagnostics, and treatment over three months.

The patient developed moderate COVID-19 three months prior to evaluation and received a 10-day course of dexamethasone, combined with nirmatrelvir/ritonavir and azithromycin. Although she initially improved, constitutional symptoms and cervical lymphadenopathy developed shortly thereafter. EBV reactivation was diagnosed one month later, and antiviral therapy with acyclovir was initiated. Despite partial symptom relief, progressive lymphadenopathy and the emergence of a supraclavicular mass led to a renewed diagnostic workup. Two months after symptom onset, tuberculosis was diagnosed, and anti-tubercular therapy began. By the end of the second treatment month, substantial clinical improvement was noted, with resolution of inflammatory markers and radiographic regression of lymphadenopathy (Figure 2).

### 2.4. Diagnostic Assessment

Initial laboratory evaluation showed leukocytosis with neutrophilia, anemia, and lymphopenia. Inflammatory markers including ESR, CRP, ferritin, procalcitonin, and D-dimer were consistently elevated, aligning with ongoing systemic inflammation. EBV reactivation was confirmed via serology (positive IgM and IgG for EBV VCA, elevated IgG to EBV EA) and PCR detection of EBV DNA (Table 1 and Table 2). Subsequent EBV testing indicated a transition to a latent phase, with IgM decline and negative EBV DNA. The PCR assays for EBV were conducted using the QuantStudio™ 5 Real-Time PCR System (Applied Biosystems, Thermo Fisher Scientific, Waltham, MA, USA), targeting the BamHI-W region for EBV DNA detection. Serological testing for EBV was performed using the Vidas^®^ EBV IgM/IgG enzyme-linked fluorescent assay (BioMérieux, Marcy-l’Étoile, France), while inflammatory markers were assessed using standard laboratory platforms such as the Cobas 6000 analyzer (Roche Diagnostics, Basel, Switzerland) for CRP and ESR, and the Siemens ADVIA^®^ 2120i Hematology System for complete blood count and D-dimer levels.

**Figure 3:**
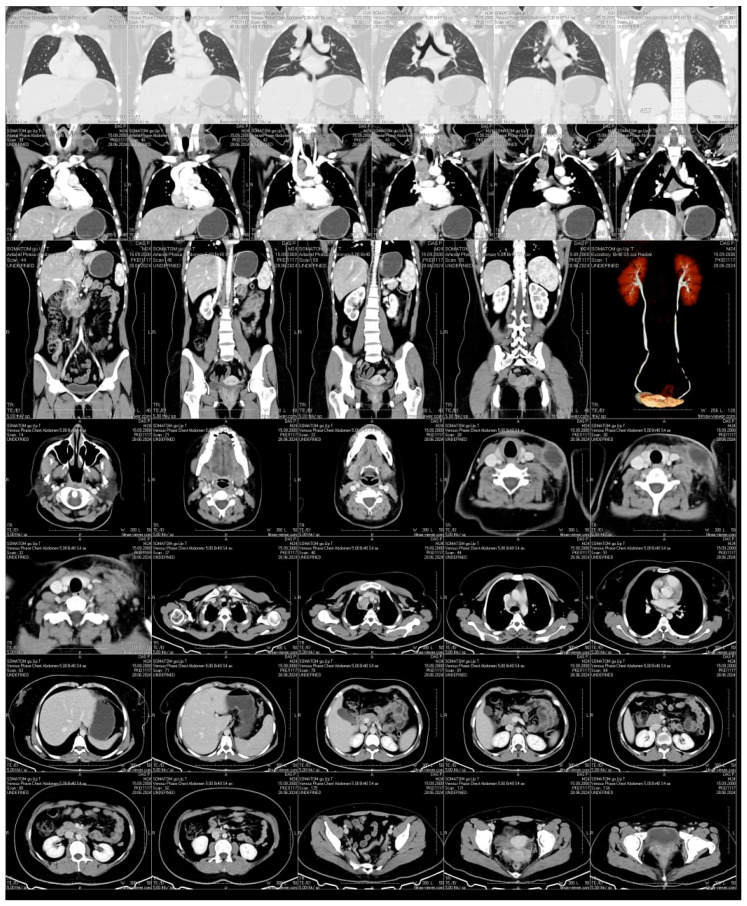
**Contrast-Enhanced CT Scan.** An encapsulated fluid collection with well-defined margins is visualized in the left supraclavicular region, consistent with an abscess or a necrotically transformed lymph node. Prominent lymphadenopathy is also observed in the paratracheal and subcarinal regions, with evidence of central necrosis. No signs of pulmonary involvement or abdominal pathology are detected.

Neck ultrasound demonstrated bilateral hypoechoic lymphadenopathy with increased vascularity. CT imaging revealed a large, encapsulated fluid collection in the left supraclavicular area, alongside enlarged paratracheal and subcarinal lymph nodes with necrotic changes (Figure 3). No pulmonary lesions or abdominal pathology were noted. Imaging was performed using a Philips iU22 ultrasound system with a high-frequency linear probe (12–15 MHz) for detailed soft tissue imaging. CT scanning was carried out on a Siemens Somatom Definition AS 64-slice CT scanner (Siemens Healthineers, Forchheim, Germany), with contrast enhancement for better visualization of the fluid collection and lymph node enlargement.

It is essential to emphasize the need for cytological and histopathological examinations, as only through these investigations was the case accurately elucidated. An extensive differential diagnosis excluded bacterial abscesses, hematologic malignancies, autoimmune disorders, and metastatic cancer. Diagnostic confirmation of tuberculous lymphadenitis was achieved through fine-needle aspiration cytology (FNAC), which showed caseating granulomatous inflammation, and PCR detection of *Mycobacterium tuberculosis*. The PCR assay targeted the IS6110 and MPB64 genes, both of which are specific for the *M. tuberculosis* complex, thereby enhancing the diagnostic accuracy. A positive IGRA, elevated ADA levels in aspirate, and positive TST further substantiated the diagnosis. Chest imaging ruled out pulmonary tuberculosis, supporting an extrapulmonary presentation. The PCR assay was conducted using a Roche LightCycler^®^ 480 System, with primers and probes specific for the IS6110 and MPB64 genes, ensuring high sensitivity and specificity. The IGRA test was performed using the QuantiFERON^®^-TB Gold Plus assay (QIAGEN, Hilden, Germany), and ADA levels were measured using the Cobas 6000 analyzer (Roche Diagnostics).

### 2.5. Therapeutic Intervention

The initial antiviral regimen for COVID-19 consisted of nirmatrelvir (300 mg) plus ritonavir (100 mg) twice daily for five days, alongside azithromycin (500 mg/day for three days) and dexamethasone (6 mg/day for ten days), following the recommendations for managing moderate COVID-19 with suspected bacterial co-infection and inflammatory complications. Following EBV confirmation, acyclovir (400 mg five times daily for ten days) was administered, consistent with CDC-recommended antiviral therapy for immunocompetent individuals with severe or prolonged symptoms of Epstein–Barr virus. Symptomatic therapy with paracetamol and ibuprofen was administered as needed.

Upon confirmation of tuberculous lymphadenitis, the patient commenced standard first-line anti-tuberculosis therapy. The intensive phase included daily isoniazid (300 mg), rifampicin (600 mg), pyrazinamide (1500 mg), and ethambutol (1200 mg) for two months, with pyridoxine supplementation (25–50 mg/day) for neuroprotection. The continuation phase (currently ongoing) consists of isoniazid and rifampicin at the same dosages for at least four additional months. Treatment adherence has been consistent, with no modifications required due to side effects. This regimen corresponds to the standardized 2HRZE/4HR protocol. Treatment adherence has remained consistent, and no modifications have been necessary due to adverse drug reactions.

### 2.6. Follow-Up and Outcomes

After two months of anti-tuberculosis therapy, the patient demonstrated substantial clinical improvement. Fatigue, fever, and night sweats resolved, and the supraclavicular mass markedly regressed. Repeat CT imaging showed reduction in lymph node size and resolution of necrotic changes. Laboratory assessments revealed normalization of inflammatory markers, restoration of hematologic values, and improved immunological parameters. Microbiological testing of aspirated lymph node material was negative for *M. tuberculosis*, indicating microbiological clearance. No adverse drug reactions were reported. Treatment tolerance was excellent, with adherence assessed through regular clinical evaluations and patient self-report.

## 3. Discussion

**Figure 4:**
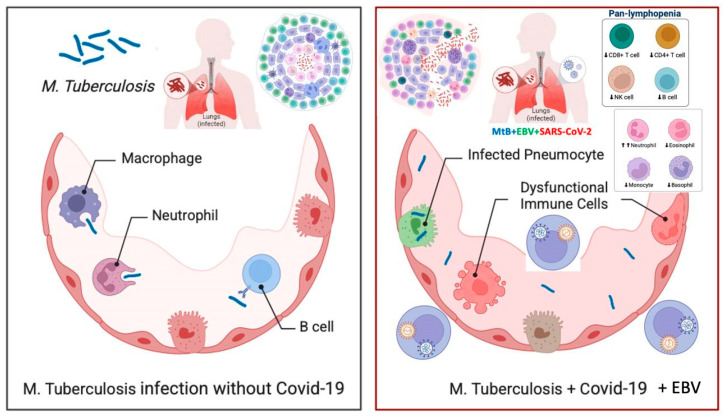
The impact of SARS-CoV-2 and Epstein–Barr Virus (EBV) co-infection on tuberculosis progression. The figure compares tuberculosis infection in the absence (**left**) and presence (**right**) of SARS-CoV-2 and EBV co-infection. The left panel illustrates an effective immune response against Mycobacterium tuberculosis (*M. tuberculosis*), where macrophages, neutrophils, and B cells help control the infection. In contrast, the right panel shows how co-infection with SARS-CoV-2 and EBV leads to immune dysfunction, including infected pneumocytes, dysregulated immune cells, and pan-lymphopenia (reduced CD4+ T cells, CD8+ T cells, NK cells, and B cells). This weakened immune defense increases the risk of tuberculosis reactivation and progression, underscoring the need for close monitoring and early intervention in affected patients.

The co-infection of SARS-CoV-2 and Epstein–Barr virus (EBV) presents a considerable immunological challenge, particularly due to its association with lymphopenia and broader implications for immune dysregulation (Figure 4) [23,24].

Lymphopenia is a well-documented consequence of both SARS-CoV-2 and EBV infections [25,26,27]. Notably, the reduction in CD4+ and CD8+ T lymphocytes impairs the host’s capacity to mount effective cellular immune responses, increasing the risk of reactivation of latent infections, including TB [28,29,30]. Host genetic predisposition may further influence immune resilience, severity of viral illness, and therapeutic outcomes. Genetic factors have been shown to modulate the likelihood of TB reactivation and determine the trajectory of post-viral immune recovery [31,32,33].

SARS-CoV-2 has been widely recognized for inducing a dysregulated immune response, often culminating in hyperinflammation or “cytokine storm” syndromes [34,35]. Elevated levels of interleukin-6 (IL-6), tumor necrosis factor-alpha (TNF-α), and interferon-gamma (IFN-γ) have been implicated in such states, contributing to systemic inflammation and subsequent immune suppression [36,37,38]. EBV, a ubiquitous herpesvirus capable of establishing latency in B cells, can similarly undermine immune homeostasis during periods of immune suppression or reactivation [39,40,41]. The synergistic effect of both viruses may extend the duration of lymphopenia and further diminish immune surveillance mechanisms, thereby promoting the progression of latent infections into active disease [42,43].

Latent tuberculosis remains a global health concern, with approximately one-quarter of the world’s population harboring dormant *M. tuberculosis* [44,45,46]. Normally, this latent state is contained through T-cell-mediated immunity and granuloma formation [47,48]. However, SARS-CoV-2 and EBV co-infection may disrupt this equilibrium [49,50,51]. Emerging evidence, including the present case, supports the hypothesis that viral infections can act as triggers for TB reactivation [52,53].

The depletion of CD4+ T cells, which play a pivotal role in granuloma formation and TB containment, further impairs the body’s ability to suppress active infection [54,55]. The compounded effects of SARS-CoV-2 and EBV on immune regulation may accelerate granuloma breakdown, progressing latent TB to an active disease state [50,56]. This highlights the need for proactive TB screening in patients with prolonged post-viral symptoms, especially in high-prevalence settings [57,58].

Given the overlapping symptomatology of COVID-19, EBV, and TB—such as fever, lymphadenopathy, and fatigue—accurate and timely differential diagnosis is essential [59,60]. Comprehensive diagnostics, including serological, molecular, and histopathological evaluation, are necessary to identify the causative agent [61,62,63]. Co-infections may obscure the clinical picture and delay treatment initiation [64,65,66], particularly in patients with comorbidities that further compromise immunity [67,68].

Identifying pharmacologic strategies to mitigate co-infection-related immune dysfunction remains a critical research priority [69,70,71,72]. Additionally, the immunosuppressive effects of corticosteroids or biologics—often used to manage severe COVID-19 or post-viral syndromes—may increase TB reactivation risk [52,73]. Optimal dosing strategies and treatment duration must be carefully evaluated, especially in individuals with prior TB exposure or sustained lymphopenia [74,75]. Alterations in gut microbiota (dysbiosis) may also influence immune regulation and disease severity, contributing to viral persistence and impaired containment of TB [76,77,78]. Comorbid conditions such as diabetes mellitus are known to worsen outcomes in both TB and COVID-19, complicating disease management [79,80,81].

The immunological interplay between SARS-CoV-2, EBV, and *M. tuberculosis* demands further investigation, particularly regarding long-term immune consequences and susceptibility to latent infection reactivation [49,82]. From a public health perspective, screening for latent TB infection in individuals recovering from severe viral illnesses may be warranted, especially in TB-endemic regions [83,84]. Early surveillance and consideration of preventive TB therapy could reduce the burden of reactivation [85,86,87]. Despite accumulating evidence, gaps remain in understanding the long-term immunologic consequences of SARS-CoV-2 and EBV co-infection. These areas require further research and integration into public health policy.

This case underscores the complexity of diagnosing and managing TB in the context of viral co-infections [60,88]. Unraveling the mechanisms of immune suppression and reactivation is essential for improving patient outcomes and reducing post-viral complications [89,90,91].

While no prior cases describing triple co-infection with COVID-19, EBV, and TB have been formally documented, existing studies support the pathophysiological plausibility and highlight the need for vigilance. EBV and COVID-19 co-infection has been linked to heightened inflammatory responses [92,93,94,95], while multiple reports have detailed the clinical consequences of TB-COVID-19 co-infection [96,97,98,99]. A systematic review of viral reactivations in COVID-19, including EBV, has further illustrated the relevance of these interactions [100].

Additional support for the possible causal relationship between COVID-19 and TB reactivation comes from previously published case reports and systematic reviews. Rawaa S. Al-kayali et al. described two patients who developed active TB after recovering from COVID-19, both presenting with persistent cough and fever—symptoms aligning with our case [101]. Moreover, a systematic review by Ayinalem Alemu et al. analyzed 33 cases from 21 studies across 13 countries, all documenting TB reactivation in COVID-19 survivors [102]. The median age of patients was 44 years (range: 13.5–80), and more than half were male. Notably, 62.5% had received corticosteroids for COVID-19 treatment. TB manifestations included pulmonary (60.6%), extrapulmonary (33.3%), and disseminated forms (6.1%). The review also found that TB onset could occur up to seven months post-COVID-19 recovery. These findings reinforce the hypothesis that immune suppression following SARS-CoV-2 infection may facilitate TB reactivation in predisposed individuals, as observed in our patient.

This case highlights how post-viral immune dysregulation may contribute to the reactivation of latent TB. Persistent lymphadenopathy and systemic inflammation, in the context of recent SARS-CoV-2 and EBV infections, required comprehensive diagnostic evaluation. Ultimately, biopsy and molecular diagnostics were key to confirming the diagnosis. Clinically, this case emphasizes the need for a high index of suspicion for TB in post-COVID-19 patients with persistent or unexplained symptoms. Future clinical care should integrate multidisciplinary collaboration to facilitate timely diagnosis and individualized management.

A limitation of this report is its single-case design. Although it offers insight into a potentially emerging clinical phenomenon, broader studies are needed to confirm these observations and deepen understanding of the underlying immune mechanisms.

## Figures and Tables

**Table 1:** Dynamics of laboratory parameters in the patient.

Parameter	Normal Range	Before Diagnosis	After Initial Treatment	Worsening Symptoms	After Tuberculosis Treatment
Hemoglobin (g/L)	120–160	118 **↓**	110 ↓	102 ↓	115 ↓
Erythrocytes (×10^9^/L)	3.8–5.2	3.7 ↓	3.5 ↓	3.2 ↓	3.6 ↓
Hematocrit (%)	36–46	35 ↓	33 ↓	31 ↓	34 ↓
Leukocytes (×10^9^/L)	4.0–9.0	9.8 ↑	11.2 ↑	12.5 ↑	10.3 ↑
Neutrophils (%)	40–75	78 ↑	82 ↑	85 ↑	76 ↑
Lymphocytes (%)	20–45	15 ↓	12 ↓	9 ↓	14 ↓
Monocytes (%)	2–10	2	1.6 ↓	1.5 ↓	1.8 ↓
Eosinophils (%)	1–6	0.5 ↓	0.5 ↓	0.2 ↓	0.8 ↓
Basophils (%)	0–1	0.5	0.3	0.2	0.4
ESR (mm/h)	<20	25 ↑	32 ↑	40 ↑	28 ↑
C-reactive protein (mg/L)	<5	12 ↑	18 ↑	24 ↑	10 ↑
Procalcitonin (ng/mL)	<0.5	0.8 ↑	1.2 ↑	1.5 ↑	0.6 ↑
D-dimer (µg/mL)	<0.5	1.5 ↑	2.3 ↑	2.8 ↑	1.1 ↑
Ferritin (ng/mL)	30–400	450 ↑	500 ↑	550 ↑	470 ↑

**Table 2:** Immunological profile changes in the patient.

Pameter	Normal Range	After Initial Treatment	Worsening Symptoms	After Tuberculosis Treatment
CD4+ T lymphocytes (%)	30–60	25 ↓	20 ↓	30
CD8+ T lymphocytes (%)	15–35	14 ↓	12 ↓	18
B lymphocytes (%)	5–20	3 ↓	2 ↓	5
NK cells (%)	5–15	4 ↓	4 ↓	6
Complement C4 (mg/dL)	18–40	10 ↓	8 ↓	15 ↓
Complement C3 (mg/dL)	90–180	110	100	130
Immunoglobulin G (IgG, Serum) (g/L)	7–16	7.0	6.8 ↓	7.8
Immunoglobulin M (IgM, Serum) (g/L)	0.4–2.3	0.6	0.8	0.7
Immunoglobulin A (IgA, Serum) (g/L)	0.7–4.0	1.1	0.9	1.3
Immunoglobulin E (IgE, Total, Serum) (IU/mL)	<100	80	78	73

## Data Availability

Data are contained within the article.
